# Is Dyslexia a Brain Disorder?

**DOI:** 10.3390/brainsci8040061

**Published:** 2018-04-05

**Authors:** Athanassios Protopapas, Rauno Parrila

**Affiliations:** 1 Department of Special Needs Education, University of Oslo, Oslo 0318, Norway; 2 Department of Educational Studies, Macquarie University, Sydney 2109, Australia; rauno.parrila@mq.edu.au

**Keywords:** dyslexia, reading difficulty, brain, neurodevelopmental disorder, neurological disorder, neuroimaging, fMRI

## Abstract

Specific word reading difficulty, commonly termed ‘developmental dyslexia’, refers to the low end of the word reading skill distribution but is frequently considered to be a neurodevelopmental disorder. This term implies that brain development is thought to be disrupted, resulting in an abnormal and dysfunctional brain. We take issue with this view, pointing out that there is no evidence of any obvious neurological abnormality in the vast majority of cases of word reading difficulty cases. The available relevant evidence from neuroimaging studies consists almost entirely of correlational and group-differences studies. However, differences in brains are certain to exist whenever differences in behavior exist, including differences in ability and performance. Therefore, findings of brain differences do not constitute evidence for abnormality; rather, they simply document the neural substrate of the behavioral differences. We suggest that dyslexia is best viewed as one of many expressions of ordinary ubiquitous individual differences in normal developmental outcomes. Thus, terms such as “dysfunctional” or “abnormal” are not justified when referring to the brains of persons with dyslexia.

## 1. Introduction

Little Johnny was in distress. He had been taking singing lessons for a few years already, but obviously, this wasn’t working for him. Every time he tried to sing he could see others cringe. He could hear the choir sound better when he remained silent and only opened and closed his mouth, but he didn’t know what else he could do about it. His highly musical family was gravely concerned: Everyone else was an accomplished singer or on the way to becoming one, but for the life of him, Johnny just couldn’t sing in tune. He was taken to a specialist who ran a whole load of tests, asked him to sing various notes and melodies, tap rhythms, and read some music; overall, quite an ordeal. Then the specialist said Johnny should not worry because it was not his fault. His brain was just miswired because something had gone wrong when it was put together, before he was born. Johnny was diagnosed with a disorder and was prescribed intervention to tackle his disability. After a few years of arduous daily training sessions, he could sing along the Christmas carols barely in tune with the rest of the family as long as he kept the volume low. Quite an accomplishment, relatively speaking. But as he was growing up, nagging questions remained: How did the specialist know there was something wrong with his brain, if she never looked inside it? And how could there be something wrong with his brain, without affecting anything else? After all, he was a perfectly normal, well-adjusted boy, with lots of friends and interests, doing fine at school, participating in all family and community activities (save the choir). Had it not been for the singing lessons, nobody would have ever come up with the idea that anything might be wrong with him.

The story of fictional Johnny probably sounds a bit far-fetched. But, unfortunately, this story has already become all but commonplace. All it takes is to substitute “reading” for “singing” and Johnny’s ordeal becomes all too familiar. But literacy and all the benefits and requirements that are associated with it have become so ingrained in our society that it is difficult to see things clearly when reading is discussed directly. We hope the singing vignette may serve to clarify some arguments, as a hypothetical comparison case to keep in mind, free from interference from established modes of thinking regarding literacy (but perhaps tainted by established notions of a “beautiful voice”). The contrast is stark: On the one hand, being able to learn to read easily and well is thought to be normal and expected, and difficulty learning to read is considered a disorder. On the other hand, being able to learn to sing easily and well is thought to be a gift, and difficulty in learning to sing is entirely unremarkable. Is there really a well-established difference regarding individual differences in propensity to acquire skill in these two domains of human culture?

The present contribution concerns persistent difficulty learning to read, commonly known as “developmental dyslexia” (among other designations). In particular, it is about the *brains* of persons with dyslexia. We were motivated by what we see as an alarming trend to consider dyslexia to be a “neurodevelopmental disorder” (e.g., [1,2,3,4,5,6]; it is even mentioned in the scope of the Journal of Neurodevelopmental Disorders). This implies that dyslexia is caused by something literally having gone wrong in the development of the brain. Some researchers have even gone so far as to call dyslexia a “neurological disorder” (e.g., [7], p. 1383; [8], p. 720; [9], p. 2453). To the extent that this reflects an underlying conceptual trend, rather than simply sloppy terminology, we believe that certain clarifications are in order.

Brains were not made for reading, or for singing. If one cannot learn to read or sing, that is not in itself evidence that there is something wrong (abnormal, dysfunctional, atypical) with their brain. To ascertain that a neurodevelopmental disorder exists we need independent evidence at the level of the brain—not just a correlate of the poor reading, or singing, but direct specific evidence of neural anatomical and functional aberration.

In the present paper, we explain what it means for something to be a neurodevelopmental or neurological disorder and what kinds of evidence would be required to justify these terms. We briefly discuss what kinds of evidence are actually available on dyslexia and what they imply. We then discuss more broadly what we believe is the larger issue, namely the reconciliation of a “biological” outlook on dyslexia, on the one hand, with a conceptualization of dyslexia as the lower end of the distribution of word reading skills, on the other. We will show that there is, in fact, no clash between these two contemporary views; rather, they are fully compatible with each other and both are well justified given our present understanding and the available empirical evidence. In contrast, the argument that dyslexia is a neurodevelopmental disorder is not justified given the current evidence. We suggest that this argument has unfortunate consequences in guiding explanatory models, research, and sometimes clinical practice towards solutions that are unlikely to meet the needs of individuals with dyslexia.

## 2. What Is Dyslexia?

Dyslexia is considered to be the most common learning disability [10], affecting children across languages, writing systems, and educational approaches. Perhaps the most widely cited definition of dyslexia states that “[Dyslexia is] a disorder manifested by difficulty in learning to read despite conventional instruction, adequate intelligence and sociocultural opportunity. It is dependent upon fundamental cognitive disabilities which are frequently of constitutional origin” ([11], as cited in [12], p. 15). More recent definitions also make the point that dyslexia is in some sense of constitutional origin. For example, the definition of the British Dyslexia Association [13] begins with “Dyslexia is a specific learning difficulty that mainly affects the development of literacy and language-related skills. It is likely to be present at birth and to be life-long in its effects.” In turn, the International Dyslexia Association [14] starts its definition with “Dyslexia is a specific learning disability that is neurobiological in origin. It is characterized by difficulties with accurate and/or fluent word recognition and by poor spelling and decoding abilities.” One main goal of the present contribution is to dispel the common misconception that equates “constitutional origin” with “faulty brain.”

Attempting to integrate educational and psychological literature, we recently proposed [15] that dyslexia concerns “a persistent and unexpected difficulty in developing age- and experience-appropriate word reading skills” (p. 333). In this definition, “word reading skills” include both accuracy and efficiency, while “difficulty” includes both very low performance as well as performance that is not exceptionally low but can only be maintained with extraordinary effort. Consistent with our current understanding of the psychology of reading, reference to “word reading skills” excludes difficulties with reading comprehension. Such difficulties may or may not accompany difficulties in word reading accuracy and fluency; if they do, they are either of a distinct (language) origin or secondary to word reading. By “persistent” we mean to exclude transient problems that may be remedied, for example, by an alternative instructional method. Finally, the term “unexpected” is meant to exclude cases in which the difficulty can be attributed to causes such as poor general cognitive ability, problems with sensory perception, or inadequate educational opportunities. Dyslexia does not refer to just anyone who cannot read, but only to those who find it very difficult to learn to read in spite of sufficient and appropriate instruction.

Notably, we see no need for additional inclusion criteria. For example, even though the great majority of individuals with dyslexia exhibit low performance in phonological awareness and rapid naming tasks (e.g., [16,17,18,19]; but there are exceptions: see, e.g., [20,21,22,23]), a “phonological deficit” is not included in our definition because (a) it is redundant when present, since the difficulty with reading skills suffices; and (b) it incorrectly excludes those few who have unexpectedly poor word-level reading skills without concomitant phonological difficulties (cf. [24]).

Our definition is in line with current conceptualizations of the term dyslexia as implying no qualitative distinction but merely referring to the low end of the reading ability spectrum, with an arbitrary cutoff [25]. The arbitrariness of the cutoff, which is usually set by statistical (and possibly financial) considerations, implies that estimates of the prevalence of dyslexia are meaningless because they are in essence defined by the criterion itself. For example, if we consider dyslexia to concern reading skills one standard deviation lower than the mean, then we are defining 16% of the population as having dyslexia; if we consider dyslexia to refer to the 5th percentile of the reading skill then we are defining 5% of the population to be dyslexic; and so on. However, prevalence estimates are often mentioned in the dyslexia literature, giving the false impression that there are absolute criteria on the basis of which dyslexia is defined, further giving rise to the expectation that such criteria might be linked to specific, potentially identifiable causal factors, whereas in fact there is nothing but a continuous distribution of reading skill, with an enormous range of individual differences.

The emerging consensus on this issue is clearly reflected in the writings of prominent dyslexia researchers over recent years. For example, Snowling [26] stated that “Dyslexia is just another name for poor reading… Where you put the cut off between dyslexia and normal reading has to be agreed within your education system, your school, it could be a national policy, a policy within a local authority, there isn’t any gold standard” (“Untangling dyslexia”). Ahmed, Wagner, and Thatcher Kantor [27] noted that “dyslexia represents the low end of normal variation in reading ability where word-level reading is the key weakness” (p. 210). Rayner, Pollatsek, Ashby, and Clifton [28] pointed out that “there is little research to support the common perception that the reading problems of dyslexic children are categorically different from those of other children who struggle with learning to read” (p. 357) and “the continuous nature of dyslexia can make it seem like a fuzzy concept, as there is no absolute list of symptoms beyond a marked difficulty with decoding and encoding written language” (p. 358). Peterson and Pennington [25] approvingly cited older sources to state that “[d]yslexia is mainly defined as the low end of a normal distribution of word reading ability” (p. 285). Similarly, Seidenberg [29] proclaimed that “[d]yslexics are children (and, later, adults) whose reading is at the low end of a normal distribution” (p. 156). Finally, in their recent comprehensive review, Elliot and Grigorenko [30] discussed various approaches to defining specific subgroups of poor readers as dyslexics, finding them all wanting for theoretical and/or practical reasons, and concluded by pointing out “[the position] commonly held by reading researchers whereby dyslexia is defined by one’s position at the tail end of the distribution curve of scores on a reading test” (pp. 173–174).

Thus, from the field of dyslexia and reading research, it seems clear that the persistent and unexpected difficulty with word reading that we commonly call dyslexia is not seen as a qualitatively distinct condition but, rather, a designation (label) for the low end of the distribution of word-level reading skills.

## 3. Neurodevelopmental Disorders

In stark contrast to the view from the reading field, the notion of a “neurodevelopmental disorder” has a distinctly qualitative feel to it, and is quite often associated, implicitly or even explicitly, with a “medical condition” or “psychiatric condition” or “psychopathology.” Indeed, dyslexia (termed “reading disorder,” as one of the neurodevelopmental disorders) is included in the diagnostic classification system used by psychiatrists worldwide (the Diagnostic and Statistical Manual or DSM, 5th edition [1]), thus implying that dyslexia can be legitimately viewed as a mental health issue. By analogy, poor little Johnny may have to consult a psychiatrist or other medical professionals to have his “singing disorder” properly identified. Indeed, by this rationale, anyone falling below some performance threshold on any behavioral domain might be taken to qualify as a mental health patient, suffering from a psychiatric disorder.

This is probably not the intended implication of including “reading disorder” in the DSM. Rather, one hopes that the inclusion is meant as a practical means to ensure that persons needing additional support in order to become functionally literate can obtain access to the corresponding assessment and remediation services. Indeed, given the potentially devastating consequences of inadequate reading skills, not limited to poor academic performance but also encompassing emotional and social outcomes (e.g., [31,32]), it is critical that reading difficulties are fully addressed, and hopefully resolved, in a timely and comprehensive manner, and that appropriate systems must be in place to take on and carry out this task as effectively and efficiently as possible. However, it is not clear to us that this purpose is best served by positing an empirically unjustified brain defect as the cause of the difficulty, which is what calling dyslexia a neurodevelopmental disorder amounts to.

The notion of a neurodevelopmental disorder seems to be a hodgepodge of conditions that become evident at a young age (infancy or childhood) and that presumably can be attributed to disrupted brain development [3,5,6], ranging from clear-cut cases with known etiologies and/or pathologies (e.g., Down, Fragile X, Tourette’s, fetal alcohol, and gene deletion syndromes; [2,33,34]), through cases with likely (if undiscovered) underlying brain pathologies (e.g., schizophrenia and autism), all the way to cases with no actual discernible neural dysfunction beyond the defining “symptoms” (such as developmental language delay and specific learning disabilities, including dyslexia). In fact, as Bishop [2,3] has pointed out, the term “neurodevelopmental disorder” has been used in different ways, encompassing both known and presumed etiologies, the common thread concerning (documented or presumed) impairment in neural development.

It is important to be very clear about the conceptual consequences of terminology. Referring back to poor Johnny, it is one thing to say “you cannot sing well because the way your brain is wired (by genetics and development) makes it very hard for you to learn to sing;” and it is a very different thing to say that “you cannot sing well because something in your brain is faulty and that prevents you from learning to sing.” In the former conceptualization, which foregrounds individual differences across levels of description (genetic, neural, and behavioral), there is nothing wrong in the brain. It is just that different brains are differentially suited to different tasks. Performance on any single task has no general implications on the health of the individual (or their brain). If it is important for a family that all its members sing well, then some of them will require a lot of extra lessons and will probably never be on par with the rest. The family must take up the cost and effort associated with helping the less able members participate in their singing activities.

In the latter conceptualization, which attributes behavioral difficulties to frank neural impairments, there is something literally wrong in the brain, something that should objectively be otherwise. In other words, Johnny’s brain has not developed properly, regardless of any particular requirements. Teaching him to sing has simply revealed the anomaly, that is, he should still be considered to have an abnormal brain if he had been brought up in a nonsinging family, in the absence of any behavioral deficiency. Although there is absolutely no difference between the two approaches in the need for specific behavioral intervention, there is a huge difference in research implications, in the perceived need for medical interventions, and, more generally, in how the individuals with difficulties are conceptualized.

Turning to the dictionaries for a bit of terminological help, we see that the notion of “disorder” refers to “an abnormal physical or mental condition” [35] or “an illness that disrupts normal physical or mental functions” [36]. Although “abnormal” can simply be taken to mean “deviating from the normal average” [37] or “deviating from what is normal or usual” [38], this is typically taken to mean “an unwelcome or problematic way” [37], or “undesirable or worrying” [38].

Therefore, taken together with the notion of “disrupted neurodevelopment,” the possible meaning of “illness”, and the lumping with conditions in which there are clear-cut neural pathologies, usage of the term “neurodevelopmental disorder” for dyslexia may reasonably be taken to imply that there is something wrong in the brain; something that, if we had the right kind of knowledge and technology, we would be able to literally point to and see, either in the mature brain or during the development of the nervous system, and probably both. In fact, the nonchalant usage of the term “neurological disorder” (which means “disease of the nervous system” [39,40]) implies one’s near certainty that nervous system pathology causes dyslexia. In other words, dyslexia can be justifiably perceived to be some kind of brain disease. But is there any evidence for that?

## 4. Brain Anatomy and Structure in Dyslexia

There is a bit of—old but famous—evidence consistent with the idea that there is something wrong in the brain of some persons who have persistent unexpected difficulty in learning to read. Specifically, Galaburda and colleagues [41,42] reported a number of anatomical anomalies in the brains of a few persons with reading difficulties. Although those findings are intriguing, they are far from decisive. The samples were extremely small (a total of 4 men and 3 women) and included participants with evidence for neurological or psychiatric conditions and participants with impairments not limited to written language, who should have been excluded from a study purporting to examine brain correlates of dyslexia. The control group was also very small, effectively precluding reliable estimation of the specificity of any findings. In other words, we do not know how frequently similar kinds of anatomical aberrations are found in the general population and what proportion of brains with such findings would come from people with reading problems. Importantly, the findings were not the same between the two studies. Contrary to popular interpretation, this actually means that the studies failed to replicate each other, and therefore failed to support the idea that any particular kind of brain anomaly is specifically associated with dyslexia. Finally, there is no evidence in the published reports that the studies were blind to the examiners, allowing the possibility of bias.

In all, this evidential basis of very few, not entirely well-selected individuals, with inconsistent and unreplicated anatomical findings of unknown prevalence in the general population, is extremely weak and certainly insufficient to support the popular contention that frank brain pathology accounts for the thousands upon thousands of children with difficulty learning to read. Although it may be interesting for researchers that frank neurodevelopmental aberrations causing various neurological conditions in some specific cases may also be associated with difficulty learning to read, this is very definitely not a firm basis on which to argue about the brain bases of dyslexia. For that we would need much larger samples of persons with severe difficulties limited to learning to read, in the context of even larger samples of brains of good readers, to establish specificity.

Although more informative anatomical studies are lacking, there have been several neuroimaging studies examining the structure of the brain (see reviews in [43,44]). In the most recent review of studies looking for structural differences between the brains of persons with dyslexia and typically developing readers, Ramus and colleagues [43] concluded that most studies were too small and the results too inconsistent to provide a clear answer, given that larger studies and meta-analyses have revealed many important limitations. They noted that there is an “apparent consensus” to the effect that the neuroanatomy of dyslexia has supposedly established the existence of specific localized structural disruptions. Ramus and colleagues attributed this viewpoint to “selective reviews of the evidence, with a marked preference for results that seem convergent” (p. 435). In fact, their own review revealed major inconsistencies and severe methodological limitations. Among the few reliable findings, a difference in total brain volume emerged most prominently, followed by a difference in the asymmetry of a brain region called the planum temporale, which, however, was only found for dyslexic boys, not for girls.

These better-established findings are very different from the localized and specific “anomalies” much of the literature would have us believe. Moreover, they are of so low specificity as to be nearly useless as “neural markers” of dyslexia. In particular, the surface of planum temporale follows the “typical” asymmetrical pattern (larger in the left than in the right hemisphere) in about 65% of the general population ([45], cited in [43]), leaving a 35% of the population with an “atypical” pattern. Meanwhile, Altarelli and colleagues [46] found an “atypical” planum temporale asymmetry (i.e., larger on the right) only in a subgroup (60%) of dyslexic boys and a minority (33%) of dyslexic girls (the same as in the general population). Therefore, even the celebrated case of the planum temporale is, in fact, a far cry from the sought-after anatomical marker of dyslexia. Given that an asymmetrical planum temporale was reported for all of the original brains of Galaburda and colleagues [41], the recent findings underscore the importance of conducting larger-scale studies on well-defined populations rather than relying on the original anatomical observations of those few special brains.

More recent studies have also hinted at other structural brain differences between groups of participants with and without reading difficulties, such as differences in average cortical regions in certain areas. Beyond important methodological concerns regarding these studies [43], there are much more fundamental problems associated with the interpretation of group differences, which do not permit any conclusions regarding their status as documenting “brain anomalies”. This is a more general issue, discussed in detail later, as it concerns both anatomical and functional studies.

## 5. Functional Neuroimaging of Dyslexia

What about functional markers? There have been several neuroimaging studies of dyslexia using functional magnetic resonance imaging (fMRI), in which groups of participants with reading difficulties are compared to groups of participants with no difficulties. The results have been summarized in recent reviews (e.g., [47,48]; see also [49,50,51,52,53]). The actual picture from the individual research papers is much noisier than the brief summaries in the reviews might lead one to assume, with many unreliable findings, unreplicated across studies, most likely due to the typically small sample sizes and, possibly, in part due to differences between tasks, sampling criteria, languages, equipment, and statistical analysis software. If one examines the actual results reported in the original neuroimaging studies, it becomes immediately obvious that patterns of neural activation differences diverge markedly across studies, not limited to precise localization of common clusters but concerning entire patterns of clusters, including major ones, extending over different gyri, lobes, and, occasionally, hemispheres (especially if one considers studies using different tasks, such as reading vs. phonological awareness, or outcome criteria for the localization, such as response to intervention).

This is not something that has gone unnoticed in the neuroimaging community. It is no accident that in their recent meta-analysis of functional neuroimaging studies of dyslexia, Martin and colleagues [51] included two tables of “convergence across studies,” one for “deep” and one for “shallow” orthographies. Those tables list the number of studies in which differences between groups with and without dyslexia were found in specific brain regions. Not surprisingly, the most consistent findings concerned the left occipitotemporal cortex, which includes the so-called “visual word form area”, thought to be critical for reading [54,55]. Out of 14 studies in each group of orthographies, there were 9 in one group and 8 in the other that reported underactivation in this area. That is, the brain area in which the best-established activation differences related to dyslexia are found, the area that is typically mentioned in reviews as a bullet-proof finding concerning dyslexia in general, was in fact identified in just 64% and 57%, respectively, of the functional neuroimaging studies that were meta-analyzed by Martin and colleagues [51]. The inferior parietal lobule came in a close second, with 7 and 8 studies, respectively, whereas other areas, some of which are also discussed in reviews as representing common knowledge, showed much lower consistency (2–5 studies out of 14). There were also a host of areas reported only in individual studies.

One important problem plaguing functional neuroimaging studies concerns sample size. Simply, most fMRI studies have too small samples to permit reliable conclusions to be drawn [56,57]. This is not specific to the study of dyslexia, of course, and is in practice difficult to address, primarily for financial reasons. But it is an issue that must be taken into account when trying to understand what the evidence actually supports. The current state of the evidence does not permit much confidence in declaring any pattern of differences as constituting the “neuroanatomical signature” of dyslexia. Whether a sufficiently consistent pattern does eventually emerge or not is something that better-powered studies can reveal in the future. A crucial obstacle that is underappreciated outside the neuroimaging field is that meta-analyses cannot help detect weak but consistent patterns from the individual studies because fMRI meta-analyses are performed on the basis of the reported cluster peaks, which have survived statistical criteria on the small samples of the original studies, thus losing all subthreshold information that would be necessary to detect weaker patterns across studies. This is an inherent problem in fMRI reporting and can only be rectified when unthresholded maps are made available by researchers instead of just the peaks of statistically significant clusters.

Still, some relatively consistent patterns do seem to recur amidst this sea of idiosyncratic reports, such as the aforementioned underactivation in the occipitotemporal cortex. Researchers must now sort out which activation differences may correspond to difficulty in learning to read, being observable prior to reading instruction, and which differences merely reflect the limited reading experience or poor reading skill. This can only be determined in comparison of findings between children and adults (see, e.g., [58]) and, in particular, in studies of the brains of pre-reading children who will subsequently encounter difficulties learning to read [52].

It seems possible that future studies, with larger and well-controlled samples, may eventually corroborate a dominant pattern of differences, or perhaps a set of commonly seen patterns. We will not summarize aspects of these patterns here, not only because they are not conclusively demonstrated, but primarily because, as it turns out, it does not matter for present purposes. All we want to point out is that all relevant findings are correlational, and typically concern group differences. That is, a group of participants with reading difficulties exhibit, on average, a higher or lower level of neural activity or neural coordination (as indicated by changes in levels of blood oxygenation causing measurable differences in the fMRI BOLD signal) than a group of participants without reading difficulties while carrying out some (usually reading-related) task(s). This is undeniable. As we explain below, it is also both unremarkable and mostly uninformative. That is, the existence of group differences does not, in fact, demonstrate brain “abnormalities” or even brain “dysfunction”. But before we get to that point we need to discuss another important problem concerning the interpretation of neuroimaging findings, one that is rarely mentioned in published studies although it affects them all.

In a typical fMRI study, one records a signal that is indirectly associated with neural activity in each volume element in the brain. This is then statistically compared between times of engagement with an experimental task, such as reading or rhyming, and times of engagement in a “control” task, such as looking at a fixation cross. The statistical comparison is first performed for each participant at each location, and then the differences found in individual participants are taken to the group level to identify locations of differences between tasks that are consistent across participants. The group-level analysis reported in individual studies is thus designed to reveal locations with consistent differences. This is very important for interpretation because “consistent” activation is not the same as “typical” activation.

In particular, it is entirely plausible that each person has a distinct pattern of activation, which includes a few areas that are consistent across participants and several areas that are not shared with many other participants. In this scenario, the group result, which is the only one reported, will reveal the consistent areas even if they exhibit only minor activation effects compared with other areas within any given person. This means that the way fMRI results are usually analyzed and reported may result in reliable findings that are not, in fact, typical, in the sense that individual participants may not exhibit the pattern of activation that is reported as group average (as seems to be the case for memory retrieval, for example [59,60]). This analytical approach is based on the assumption of consistent localization across participants, which is a theoretical prerequisite to group analysis in fMRI. The fragility of this assumption is well known to fMRI researchers, who have debated alternative approaches (see, e.g., [61] and the first four chapters in [62]).

How is this relevant to understanding the brain basis of dyslexia? Well, if we do not know the variability in individual patterns of activation underlying reading and reading-related tasks, then we have no evidential basis for the “typical” pattern against which other patterns can be compared and potentially found to be “atypical.” The very notion of typicality hinges on an understanding of individual differences, that is, of the extent and magnitude of variability across individuals, such that certain features or patterns are documented to be common (hence “typical”) whereas others occur relatively infrequently (hence are “atypical”). The group averages typically reported in neuroimaging studies are entirely moot with respect to typicality. However, studies of patterns of activation at the level of individual brains are few and far between (e.g., [59,60]), and as far as we know they do not include any studies on reading.

**Figure 1:**
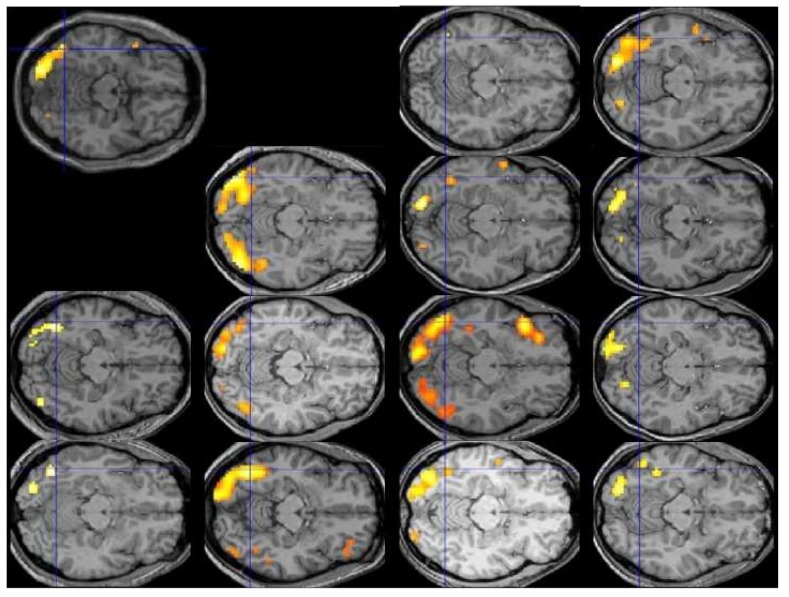
Results of individual-subject analysis (1st level) and group analysis (2nd level; top left image) for reading vs. fixation in SPM5 using data from the first 13 participants volunteering for the study of Protopapas et al. [63]. Significant activation (*p* < 0.001, uncorrected) for the contrast [0.5 × words + 0.5 × pseudowords − fixation] is shown on horizontal slice z = −14 of individual participants’ own normalized and coregistered structural (T1) images, and on SPM8 (Wellcome Trust Centre for Neuroimaging, London, UK; http://www.fil.ion.ucl.ac.uk/spm/software/spm8/) default for the group result. Blue crosshair at x = −45 y = −64 in all images. See [63] for details on methods.

Lest the reader be inclined to dismiss this admonition as implausible or overly pedantic, Figure 1 displays the individual patterns of activation for the contrast of reading vs. fixation in the first 13 participants of Protopapas and colleagues [63], all analyzed and displayed using exactly the same procedure and criteria, along with their group average (top left). The crosshair centers on the group local maximum, within the visual word form area, which is the most robustly activated area in this sort of contrast across studies and languages. We would be hard-pressed to say what the typical pattern is by looking at the individual images, despite the robustness of the group finding. In fact, we might be tempted to consider some of these participants to be atypical, especially with respect to the group result, even though none of them had any reading difficulties.

Analysis of activation patterns at the level of individual brains, performed over large representative samples, is the only way to find out what is typical, if typical patterns do in fact emerge, and how large deviations from them are uncommon enough (by some arbitrary criteria) to be considered truly atypical, before the typical and atypical patterns can be related to behavioral manifestations such as good vs. poor reading. Unfortunately, the current state of the art in functional neuroimaging does not permit any judgments regarding typicality of activation, much less proclamation of any patterns (or individual brains) as “atypical”. It should then be clear by now that the existing fMRI findings, suggesting that the areas most consistently activated among a group of good readers are not entirely identical with the areas most consistently activated among a group of poor readers, do not imply that any of the good or any of the poor readers do in fact exhibit overall similar or different patterns of activation, typical or atypical.

## 6. What Do Brain Differences Imply?

In the contemporary context of looking to neuroscience hoping to find the material basis of psychology (and sometimes, perhaps inappropriately, education; see [64]), it seems self-evident, even trite, to say that every aspect of our behavior and our internal mental experience can be safely ascribed to some aspect of brain function. There is little doubt that it is the function of the brain that constitutes every thought, every action, every intention, and every feeling. What else could it be? When we speak, it is the function of the brain that does the speaking and the thinking and the planning underlying it. Similarly, when we read, it is the function of the brain that constitutes the perceptual and cognitive processes amounting to the act of reading. This pervasive—if not always explicit—conceptual substrate is the reason why we look to the brain hoping to find clues that may help us understand what we have traditionally described in terms of cognitive and psychological theories.

Given this understanding, it also becomes self-evident to go on to state that any differences in performing a task, any differences in capacities, skills, or abilities, must necessarily correspond and amount to differences in the brain (or brains) that carry out the tasks, whether within a person (across time) or between persons. Quite simply, there is no other possible source for the differences: If two persons achieve different levels of performance in a task, this directly and literally implies that the brains of the two persons must be different in precisely the right way and extent that accounts for the observed performance difference. That is, if two persons are differentially efficient in acquiring a skill, this means that their brains differ in such a way that one of the brains is more efficient than the other in modifying itself to adapt to the skill through training. If a person learns a task very easily, this means that the way their brain is set up at the time of learning is such that carrying out this particular task has large, lasting effects that facilitate processing in subsequent encounters with the same specific task. In contrast, if a person learns a task with difficulty or not at all, this means that the way their brain is set up is such that attempts to carry out the task are not met with similar success but result in relatively little (or no) benefit toward future encounters. Unless something truly extraordinary forces us to abandon our entire contemporary outlook on brain function as the material basis of all cognitive function, this is an inescapable, self-evident account of all sorts of performance differences.

That performance differences exist between individuals is hardly newsworthy. Individual differences are rampant in every aspect of our existence, physical as well as psychological, including cognitive. Whatever skill we look at, we find that different people are differentially effective (or efficient) in learning it. Some learn to sing easily and eventually do it so well that they become acclaimed singers; others cannot carry a tune to save their life, just like our fictional little Johnny. Some take to sports with dexterity and stamina, on their way to becoming accomplished athletes; others can be clumsy beyond salvation. Some dance with grace while others (present authors included) are painful to watch; some can find their way in the woods while others get lost in their own neighborhood; some can solve Rubik’s cube, play competitive chess, or find fractions and geometry easy and pleasant; the list is endless.

Obviously, some people learn to read easily and well and others struggle with it. There is nothing remarkable about that. The only difference is that being hopelessly out of tune, or clumsy, or unable to find one’s way on a map or in a mall, isn’t limiting one’s prospects in contemporary society nearly as much as being illiterate is. In other words, it is the exigencies of modern society, with its demand for universal literacy, that make reading stand out in this list of artificial sociocultural domains, in the sense that those whose brains are somehow less well suited to the task of learning to read are given additional support (and pressure) to bring their achievement within what is considered a sufficiently functional level. Because so much is based on written language in the modern literate society, it is quite appropriate that structures and procedures exist to detect and support those who need more than regular instruction to become functionally literate, to the extent possible.

The main (if trivial) point here is that the behaviorally observed differences in ability to learn to read, and in eventual reading and spelling skills, are an expression of differences in brains. This is something we know to be the case without looking at anyone’s brain, because there is nothing but the brain that can be responsible for learning to read or for differences in learning to read. In this sense, merely finding that there are differences between the brains of those who read well and those who don’t is not just unremarkable; it is not simply expected; rather, it is essentially certain a priori. In fact, if no differences were to be found this would mean that our methods are not good enough, or that we are not looking at the brain in the right way, or that our technology is not yet sufficiently developed to detect the differences (cf. [65]). Therefore there is nothing to celebrate when brain differences are found. The only possibly interesting aspects of such findings are the details of the differences: what kind, where, and how they affect brain function with respect to the task under investigation.

Put in other words, finding differences in brains does not lead to the conclusion that “dyslexia is biological” because dyslexia could not have been anything but “biological” to begin with, even before any brain research had been undertaken. Reading itself is biological in this sense. Finding differences in brains merely implies that our current technology is sufficiently advanced to begin to dissect the systematic structural and functional differences between brains that make them differentially amenable to learning to read (or do anything else).

## 7. What Can Group Differences Show?

So far this leaves us with the following elements: Dyslexia is the low end of the continuous distribution of word-level reading skills, and differences in skill arise due to differences in brains. Therefore there must be differences between the brains of persons with dyslexia and those with no difficulty learning to read. And, in this sense, dyslexia is “biological”, just like any other difference in skill, ability, propensity, attitude, etc. Studies comparing groups of people differing in reading skills have confirmed this (see reviews in [43,44,47,48,51,58,66]). Studies comparing groups of people differing in other kinds of skills have also confirmed this, including, for example piano players [67], language learners [68,69], and taxi drivers [70], among others. There is little point in listing more comparisons because brain differences must necessarily exist whenever skill differences exist.

The differences need not be the same for every person in a given grouping, because there are also differences in the individual brains within each group, and, in all likelihood, many different ways to achieve each performance outcome. That is, it is possible that different brain configurations result in similarly good (or similarly poor) performance or capacity in a domain. This means that some individual differences that can be causally related to the observed performance differences are not discoverable by group comparisons. Some of the differences (either common or idiosyncratic) must be the outcome of having acquired the skill, expressing the final (or current) level of accomplishment (not only in the brain but also in other domains; cf. [71]). Yet some differences must have existed at the time of beginning to learn the skill, causing the differential effectiveness of the training or acquisition processes, justifying—in this almost tautological sense–the reference to “neurobiological” or “constitutional” origin in some definitions of dyslexia. Those brain differences should be eventually possible to detect, and maybe even understand, so it is great that researchers are beginning to tackle such issues with increasingly sophisticated methods and tools (cf. [47,52]).

However, as should be clear by now, no reported differences between groups could ever establish that dyslexia is a neurodevelopmental disorder, or a neurological or psychiatric condition. This is because differences in any aspect of brain structure or function are just the physical substrate of cognitive and performance differences, which we already know exist.

We suspect that some may use the term “neurodevelopmental disorder” to simply mean that the difficulty learning to read is a result of how the brain is made up at the time of learning (with which we concur). Nevertheless, this usage is at odds with the meaning of the term as defined in the relevant literature cited above. Specifically, the concept of neurodevelopmental disorder requires *disrupted* brain development, that is, a developmental *failure* of some sort. However, group differences in brains, no matter at what point they are observed, can never establish that anything has actually gone wrong with brain development because “different” is not the same as “wrong.” This is not idle political correctness or a philosophical musing about what “normal” means; this is an issue of both core theoretical substance and consequential practical implications. The jump from a descriptive finding of an average “difference” between groups to a normative conclusion of specific brains being essentially “wrong” is simply unjustified, as it violates core epistemological boundaries. Thus, we submit that the neuroimaging findings are commonly misinterpreted and the frequent assertions that the findings have demonstrated abnormalities are wholly unjustified and, hence, incorrect.

To show that neural development has been disrupted, licensing usage of the term “neurodevelopmental disorder,” one must go beyond the existence of mere differences between high performers and low performers (a descriptive finding). One must demonstrate that something in the way the brain has developed is not the way it “should” be (a normative statement), in a well-defined and independently justified sense; and one must also demonstrate that what is wrong is very strongly related to the observed behavioral shortcomings or failures. That is, first of all, atypical brain development must be concretely demonstrated in specific brains with appropriate methods, after quantifying and understanding the variability found in large representative samples of normal brains. Subsequently, claims of “dysfunctional” structures, networks, or patterns of activation must be specifically justified in relation to corresponding functional counterparts, again taking into account the full extent of normal variability. None of these are possible with simple group comparisons of (structural or functional) neuroimaging data or with the tiny samples so common in the neuroimaging literature.

Further, any indication of poor brain function (not just difference from another group, but demonstrably dysfunctional *at the brain level*) due to the atypical development of the brain must be specifically tied to the behavior in question. That is, a very large proportion of those with atypical brains must fall under the behavioral impairment category, and conversely, a very large proportion of those with the behavioral impairment must exhibit one or more atypical brain features, taking behavioral and neural heterogeneity into account. Importantly, for something to qualify as being wrong, that is, abnormal, in the brain, it is far from sufficient to find that it happens in, say, 10% of the population. Ten percent may be a relative minority but it is a rather sizeable minority. It may be useful to keep in mind that it has taken decades for our society to realize that left-handedness is not an ailment but just a less frequent trajectory of normal developmental routes. Let us not have to tread that course all over again.

In other words, to claim that something is wrong, we must find evidence that it is not only atypical (i.e., occurring in a small fraction of the population), but also clearly dysfunctional, that is, causing the brain to function poorly or improperly. We contend that this is very unlikely to be the case in dyslexia, not only because of the continuity with the rest of the reading skills distribution (recall the arbitrary cutoff!), but mainly because dyslexia is the manifestation of propensity in a narrow artificial domain. That is, demonstrating frank brain dysfunction in dyslexia is very unlikely because the majority of people with difficulties learning to read are in fact perfectly normal and have no other major difficulties in their lives beyond written language. They speak fine, they walk fine, they socialize fine, they work and have families and are fine members of our society. This contrasts sharply with frank neurodevelopmental disorders, such as fetal alcohol syndrome, where normal human function can be substantially and obviously compromised, limiting one’s prospects, e.g., for social interaction, in a pervasive way across contexts and situations. In contrast, the only “ailment” of dyslexics concerns their response to a human invention: They find it difficult to learn the artificial code of written language, which (unfortunately for them) has come to occupy a dominant place in modern society.

This is not to deny that other systematic group differences may be associated with dyslexia, related, for example to speech and language development [72], psychophysical performance (see discussion in [73,74]), or even various indices of neural function (with typically much weaker associations; e.g., [7,75,76]). Such differences, however scientifically informative, are far from diagnostic, as they invariably concern a minority of persons with reading difficulty and there is great overlap in performance between those with and without reading difficulty (even when the groups are well separated on measures of reading skill). Therefore, such associated group differences do not in any way constitute evidence for impairment beyond written language, even if they attest to on average somewhat lower performance in other domains, which may or may not be related to some aspects of some reading difficulties. Finally, all such findings are merely correlational and are probably most parsimoniously interpreted as reflecting common-cause situations in neural developmental trajectories or other etiological factors [77] rather than as directly related to the reading difficulties [73].

Put into perspective, calling dyslexics neurodevelopmentally disordered, and diagnosing them with disrupted neural development, amounts to calling neurodevelopmentally disordered any group that performs beyond some arbitrary cutoff on any skill. This includes, for example, little Johnny and all poor singers, the clumsy children who are poor in sport or dance, and all those who perform at some low percentile in driving, chess, or speed dating. Given that most of us have found it overly challenging to acquire at least some of the skills we have encountered in our lives, and would probably have trouble with many other skills we have yet to encounter, a consistent application of the criteria used for dyslexia across domains would literally imply that we all suffer from some kind of neurodevelopmental disorder. We hope we are not alone in finding this prospect untenable. The fact that modern society values literacy over singing, chess, or sense of direction does not make it a stronger candidate for a diagnosis of a disorder.

Moreover, consistent application of the extreme-performance criterion would also include, for exactly the same reasons, all those who are at the *top* percentiles in reading, math, singing, sport, dancing, etc. After all, if our criterion for calling a group “neurodevelopmentally disordered” is the brain difference underlying their extreme performance, there is no way to exclude the top performers: Due to their high performance, it is certain that their brains will differ from those of an average performing group in precisely those ways and extents that will account for the high performance. All that remains is to undertake the brain studies necessary for demonstrating the certain-to-exist differences. Then, applying the exact same rationale and criteria as in dyslexia, we would end up with a new diagnostic manual including “specific chess grandmaster disorder” as well as “specific mathematical excellence disorder” and so on.

## 8. Where Does that Leave Us with Dyslexia?

To sum up, there is at the moment no evidence to suggest that difficulty in learning to read words accurately and fluently is associated with anything having gone wrong in brain development. That is, dyslexia cannot be justifiably considered to be a “neurodevelopmental disorder” (much less a “neurological disorder”). This is in spite of the fact that dyslexia must be viewed as an expression of brain structure and function; that is, individual differences in normal brain development account for individual differences in the facility with which written language skills are acquired and expressed and in the levels of performance that are reached in given environments, with a given content and amount of instruction and experience. We must disentangle the two notions, as they are fully compatible: On the one hand, dyslexia is “biological” in the sense that it is brain structure and function that determines reading skill, including the highest and lowest levels of skill and all those in between. On the other hand, the low reading performance in dyslexia is not a symptom of some brain disorder, or even of disrupted neural development, but just the outcome of normal developmental trajectories that happen to be less efficient in acquiring and expressing written language skills, in the context of rampant, ubiquitous individual differences.

This view does not in any way imply that brain differences are genetically or otherwise predetermined. The outcomes of complex developmental trajectories we refer to lie at the intersection of multiple cumulative hereditary, cognitive, behavioral and environmental effects and their various and multiple interactions under each individual’s particular history of circumstances. Simply put, brains are no more predetermined than the complex cognitive skills and psychological states they constitute are. As we have previously noted [15], we submit to the contemporary view that to understand reading difficulties it is necessary to study them in their proper multi-factorial context [77,78] under a neurodevelopmental perspective ([79,80]; see also [49,81]), taking into account multiple factors that can affect the final outcome, in complex patterns of interaction, across levels of description, causal networks, and generations.

In particular, let us keep in mind that dyslexia is not a qualitatively distinct entity but just the low end of the word reading skill distribution, in continuity with the rest of the distribution, and with an arbitrary cutoff that essentially defines its prevalence on this behaviorally defined continuum. The difficulties in learning to read are naturally accompanied by differences in the average patterns of activation, connectivity, or structural properties in the brains, as they must, given that no behavioral differences can arise in the absence of neural differences. However, there is no evidence demonstrating that one end of a purported neural continuum, presumably reflecting the behavioral continuum, represents outcomes of developmental failure. In this context, to consider brains that do not lend themselves to efficiently learning to read as exhibiting “anomalies” or “abnormalities” [9,49,50,51,52,53,82] or to be “abnormal” [66], “dysfunctional” [7,58], or even “atypical” [47], without any specific evidence at the individual brain level and without even an understanding of the extent of normal variability, sounds potentially misleading, if not downright inappropriate.

In saying this we do not wish to imply that specific lesions, or frank neurodevelopmental disorders, cannot lead to reading behaviors that meet any given diagnostic criteria for dyslexia; they clearly do. However, for most individuals with difficulty learning to read there will be no identifiable anatomical or functional markers of dyslexia in their brains to set them apart from those with no reading problems. The critical issue is that the search for a single (or double or triple) cause for a behaviorally defined disorder, at any level of analysis, is misguided in its focus and counterproductive in its implications for interventions. As compartmentalized approaches are gradually giving way to multifactorial developmental models and the focus moves on to understanding individual trajectories [15], we submit that it is time to replace the expectation of some sort of brain defect with an appreciation of individual variability in normal neural development and its multi-faceted consequences across skill domains.

We have outlined above the type of research findings that would be necessary in order to establish whether any actual anomalies are in fact involved in the causal pathways to dyslexia. Although we find it unlikely that they are, this is ultimately an empirical question that should be resolved by further studies, appropriately designed. We cannot prove that dyslexia is not a neurodevelopmental disorder, and this is not what we have tried to do in this contribution. Instead, we have explained why none of the available kinds of evidence is relevant for deciding whether dyslexia is a neurodevelopmental disorder or not, and that it is at best premature to have implicitly decided that it is. We have criticized the widespread conflation of two very different issues, namely the relational descriptive claim that groups differ in their average values on some measures (brain-related or not) and the normative claim that one group is composed of brains that have failed to develop properly. The former claim is well supported by available evidence but is irrelevant to the issue in question, whereas the latter claim requires mapping of individual differences, appropriate delineation criteria, and independent demonstration of dysfunction, none of which have been forthcoming.

Therefore, we believe that the field has been too hasty in embracing a position that is not supported by evidence. Although some may see the “neurodevelopmental disorder” designation as a tactical move to ensure much-needed support resources, we believe that such concerns are unfounded. The need for interventions to remediate poor reading does not arise from the putative brain defects but from the poor reading itself, in the context of the modern literate society. Accordingly, recognizing that dyslexia does not constitute developmental failure, but is just an expression of normal individual differences, should have no practical implications for the special provisions that are needed to ensure universal literacy. By analogy, little Johnny needs a lot of extra support, singing lessons, and probably special parts in the choir, if he is ever to become an adequately functional member in the family activities. This is equally true whether or not he is told he has an unidentified brain defect.

This shift of focus can also enable us to appreciate that “dyslexia” is not, in fact, a cause of poor reading but, rather, a label for it. Dyslexia is not some pre-existing brain fault, distinct from normality, which hinders learning to read. Rather, dyslexia is just what happens when a brain is not particularly well suited to learning to read, a “shorthand descriptor [that] summarizes the…major area of deficit” ([3], p. 39). As aptly put by Bishop and Rutter [3], “[a] statement such as ‘My child can’t read because he’s dyslexic’ is not an explanation, rather it is a circular redescription of the problem” (p. 39). This is because the term “dyslexia” is merely a label for poor word reading that persists in spite of appropriate educational experiences, rather than referring to an underlying cause for it. Treating difficulty learning to read as some sort of neural disorder, akin to brain damage, is as offensive to the people who struggle with reading as it is misleading for the researchers who try to understand it and for the clinicians and educators who encounter it in their daily practice and will mistakenly come to associate it with pervasively disabling conditions with identifiable pathology and substantially poorer prospects.
